# Supplementary material for: Content-rich biological network constructed by mining PubMed abstracts
Source: BMC Bioinformatics. 2004 Oct 8;5:147. doi: 10.1186/1471-2105-5-147 (PMC528731; doi:10.1186/1471-2105-5-147)
Supplement: Additional File 2 — The original results of the above study (non-essential files are deleted to keep the file size under the limit set by BMC bioinformatics). [file 1471-2105-5-147-S2.bz2 › chilibotAdditionalFile2/dip20/769ID10633096E18160/html/FECI_FECR.html]

 


 **FECI** and **FECR** 
  
Found 19 abstracts in PubMed, retrieved 19.  
 

 What does Google say? 
 PDF only 
| .edu only 

---

**Interactive relationship** (e.g. stimulation, inhibition, etc)

**Neutral relationship**- Surface signaling in ferric citrate transport gene induction interaction of the FecA,  **FecR** , and  **FecI**  regulatory proteins.  Ref: 10633096 J Bacteriol, 2000
- It is concluded that the N terminal portion of  **FecR**  activates  **FecI** .  Ref: 7752886 Mol Microbiol, 1995
- The results demonstrate that region 4 of  **FecI**  specifically binds  **FecR** .  Ref: 12057967 J Bacteriol, 2002
- **FecR**  activates  **FecI** , and  **FecI**  activates transcription of the fecABCDE transport genes.  Ref: 8596456 Mol Microbiol, 1995
- FecA 1 79 interacts with  **FecR**  101 317 and  **FecR**  1 85 interacts with  **FecI**  1 173.  Ref: 10633096 J Bacteriol, 2000
- The data reveal properties of the  **FecI**  ECF factor distinct from those of sigma 70 and further support the novel transcription initiation model in which the cytoplasmic portion of  **FecR**  is important for  **FecI**  activity.  Ref: 11114913 J Bacteriol, 2001
- Regulation of citrate dependent iron transport of Escherichia coli  **fecR**  is required for transcription activation by  **FecI** .  Ref: 7752886 Mol Microbiol, 1995
- Interaction of N proximal regions of predicted  **FecR**  homologs with region 4 of predicted  **FecI**  homologs of Pseudomonas aeruginosa was demonstrated.  Ref: 12057967 J Bacteriol, 2002
- no interactions occurred between heterologous combinations of the P. aeruginosa proteins and between a P. aeruginosa  **FecI**  homolog and E. coli  **FecR** .  Ref: 12057967 J Bacteriol, 2002
- **FecR**  transmits the signal across the cytoplasmic membrane and activates  **FecI**  located in the cytoplasm.  Ref: 8602163 Mol Gen Genet, 1996
- In in vitro binding assays,  **FecR**  that was His tagged at the N terminus His 10  **FecR**  and bound to a Ni nitrilotriacetic acid agarose column was able to retain FecA, and  **FecR**  that was His tagged at the C terminus  **FecR**  His 6 retained  **FecI**  on the column.  Ref: 10633096 J Bacteriol, 2000
- These data clearly support a model that proposes interaction of the periplasmic N terminus of FecA with the periplasmic C terminal portion of  **FecR**  and interaction of the cytoplasmic N terminus of  **FecR**  with  **FecI** , which results in  **FecI**  activation.  Ref: 10633096 J Bacteriol, 2000
- The cytoplasmic portion of the  **FecR**  mutant proteins,  **FecR**  1 85, did not interact with wild type  **FecI** , in contrast to wild type  **FecR**  1 85, which induced  **FecI**  mediated fecB transport gene transcription.  Ref: 11114913 J Bacteriol, 2001
- FecA and beta galactosidase syntheses under transcription control of the fecB gene fecB Mu d1 were constitutive in  **fecI**  transformants and were regulated by iron III dicitrate in  **fecI**   **fecR**  transformants.  Ref: 2254251 J Bacteriol, 1990
- The signal is then transduced across the cytoplasmic membrane by the  **FecR**  protein, which in turn activates the  **FecI**  sigma factor that directs the RNA polymerase core enzyme to the fec transport gene promoter.  Ref: 9148773 Arch Microbiol, 1997
- It is postulated that FecA, TonB, ExbB and ExbD transfer the signal across the outer membrane, while the regulatory protein  **FecR**   [ **FECR** ]  transmits the signal across the cytoplasmic membrane to  **FecI**  in the cytoplasm.  Ref: 7729419 EMBO J, 1995
- An in vivo deletion analysis using a bacterial two hybrid system assigned the interaction of the  **FecR**  and  **FecI**  proteins to the cytoplasmic portion of the  **FecR**  transmembrane protein and region 4 of  **FecI** .  Ref: 12057967 J Bacteriol, 2002
- Functional interaction of region 4 of the extracytoplasmic function sigma factor  **FecI**  with the cytoplasmic portion of the  **FecR**  transmembrane protein of the Escherichia coli ferric citrate transport system.  Ref: 12057967 J Bacteriol, 2002
- We report that in vivo the chromosomally encoded  **FecI**  protein activates transcription of the fecA and fecB transport genes in response to ferric citrate and the  **FecR**  protein.  Ref: 7752886 Mol Microbiol, 1995
- Two missense mutations in region 2.1 of  **FecI** , S15A and H20E, partially restored induction of ferric citrate transport gene induction of the  **fecR**  mutants by ferric citrate.  Ref: 11114913 J Bacteriol, 2001
- **FecR**  then transmits the signal across the cytoplasmic membrane into the cytoplasm and activates the  **FecI**  sigma factor, which binds to the RNA polymerase core enzyme and directs the RNA polymerase to the promoter upstream of the fecABCDE transport genes to initiate transcription.  Ref: 12732308 Curr Opin Microbiol, 2003
- **FecR**  receives the signal in the periplasm from the outer membrane protein FecA loaded with ferric citrate, transmits the signal across the cytoplasmic membrane, and converts  **FecI**  in the cytoplasm to an active sigma factor.  Ref: 12813067 J Bacteriol, 2003
- Interaction of  **FecR**  with  **FecI**  was demonstrated by showing that isolated  **FecR**  inhibited degradation of  **FecI**  by trypsin.  Ref: 12618442 J Bacteriol, 2003
- Control of the ferric citrate transport system of Escherichia coli mutations in region 2.1 of the  **FecI**  extracytoplasmic function sigma factor suppress mutations in the  **FecR**  transmembrane regulatory protein.  Ref: 11114913 J Bacteriol, 2001
- We conclude that transcription of the fec transport genes is regulated by  **FecI** , which responds to ferric citrate via  **FecR** .  **fecI**  and  **fecR**  co transcription is inhibited by the iron loaded Fur repressor, which then results in a low level of transcription of the fec transport genes.  Ref: 8602163 Mol Gen Genet, 1996
- In addition, missense mutations in the  **fecI**  promoter region resulted in a twofold increased transcription in  **fecR**  wild type cells and a partial restoration of fec transport gene transcription in the  **fecR**  mutants.  Ref: 11114913 J Bacteriol, 2001
- Binding of  **FecI**  to beta and beta 1 313 was enhanced by  **FecR**  1 85, which represents the cytoplasmic portion of the  **FecR**  protein that transmits the inducing signal across the cytoplasmic membrane.  Ref: 12618442 J Bacteriol, 2003

**Non-interactive relationship** (e.g. studied together, co-existance, homology, etc.)

- Missense mutations randomly generated by PCR were localized to region 4 of  **FecI** , and the mutants were impaired with regard to the interaction of  **FecR**  with  **FecI**  and fecB lacZ transcription.  Ref: 12057967 J Bacteriol, 2002
- the residual activity of mutated  **FecI**  in the absence of  **FecR**  was not higher than that of wild type  **FecI** .  Ref: 11114913 J Bacteriol, 2001
- **fecI**  encodes a sigma 70 type factor that is necessary for transcription of the ferric citrate transport genes fecABCDE.  **fecR**  encodes a cytoplasmic transmembrane protein that is required for the induction of fec transport gene transcription by ferric citrate binding to the FecA outer membrane receptor protein.  Ref: 9393439 Mol Gen Genet, 1997
- PupI and PupR do not share homology with the classical bacterial two component systems but display significant similarity to the  **FecI**  and  **FecR**  proteins of Escherichia coli involved in regulation of ferric dicitrate transport.  Ref: 8026465 EMBO J, 1994
- Mobility band shift of fecA promoter DNA caused by cell lysates required growth of cells in the presence of ferric citrate and expression of FecA,  **FecI**  and  **FecR** .  Ref: 8596456 Mol Microbiol, 1995
- Additional ferric citrate specific regulatory proteins are  **FecR**  in the cytoplasmic membrane and the  **FecI**  sigma factor in the cytoplasm.  Ref: 9573190 J Bacteriol, 1998
- The level of the regulatory proteins  **FecI**  and  **FecR**  is adjusted to the intracellular iron concentration and determines the degree of the response of the cell to ferric citrate in the medium.  Ref: 9148773 Arch Microbiol, 1997
- The  **FecI**  protein was found in the membrane, and the  **FecR**  protein was found in the periplasmic fraction.  Ref: 2254251 J Bacteriol, 1990
- ii two pairs of signal transducers, one homologous to phoR phoP of B. subtilis, encoding regulators of phosphatase biosynthesis, and the second to the  **fecI**   **fecR**  of Escherichia coli, which is responsible for the regulation of the citrate dependent iron III transport system.  Ref: 7934829 Mol Microbiol, 1993
- Binding of ferric citrate to the outer membrane protein FecA initiates a signal that is transmitted by  **FecR**  across the cytoplasmic membrane into the cytoplasm where  **FecI** , the sigma factor, is activated.  Ref: 10633096 J Bacteriol, 2000
- Bound ferric citrate does not have to be transported but initiates a signal that is transmitted by FecA across the outer membrane and by  **FecR**  across the cytoplasmic membrane into the cytoplasm, where the  **FecI**  extracytoplasmic function ECF sigma factor becomes active.  Ref: 11114913 J Bacteriol, 2001
- It is proposed that the information flux from the cell surface to the cytoplasm involves a series of conformational changes of the proteins FecA,  **FecR** , and  **FecI**  in that order.  Ref: 9148773 Arch Microbiol, 1997
- Analysis of the Bacillus subtilis genome sequence revealed two open reading frames, designated sigX and ypuN now termed rsiX, that are homologous to  **fecI**  and  **fecR** , respectively, of Escherichia coli.  Ref: 9393439 Mol Gen Genet, 1997
- Analysis of the genetic region upstream of the bhu cluster identified open reading frames predicted to encode homologs of the Escherichia coli ferric citrate uptake regulators  **FecI**  and  **FecR** .  Ref: 11418569 J Bacteriol, 2001
- In the absence of citrate,  **FecR**  inactivates  **FecI** .  Ref: 2254251 J Bacteriol, 1990
- The in vivo assay involved the determination of the FecA,  **FecR** , and  **FecI**  interacting domains with the bacterial two hybrid Lex based system.  Ref: 10633096 J Bacteriol, 2000
- Growth was restored by transformation with plasmids encoding  **fecI**  or  **fecI**  and  **fecR** .  Ref: 2254251 J Bacteriol, 1990
- This binding elicits a signal that is transmitted by  **FecR**  across the cytoplasmic membrane into the cytoplasm, where the sigma factor  **FecI**  directs the RNA polymerase to the promoter upstream of the fecABCDE genes.  Ref: 12057967 J Bacteriol, 2002
- We propose that  **FecR**  spans the cytoplasmic membrane once, interacts in the periplasm with its C terminal region with FecA occupied by ferric citrate, and transmits the information through the cytoplasmic membrane into the cytoplasm, where it converts  **FecI**  into an active sigma factor.  Ref: 9573190 J Bacteriol, 1998
- Two genes, termed  **fecI**  and  **fecR** , that mediate induction by iron III dicitrate have been identified upstream of fecA.  Ref: 2254251 J Bacteriol, 1990
- The promoter of the regulatory genes  **fecI**  and  **fecR**  is not controlled by ferric citrate but is regulated by iron via the Fur repressor.  Ref: 9148773 Arch Microbiol, 1997
- Therefore, transcription of the fec transport genes is subjected to double control cells first detect iron deficiency and respond by synthesis of the regulatory proteins  **FecI**  and  **FecR** , which initiate transcription of the fec transport genes, provided ferric citrate is available.  Ref: 12732308 Curr Opin Microbiol, 2003
- The synthesis of the outer membrane receptor PupB, involved in the transport of the ferric pseudobactins BN7 and BN8, is induced by the ferric siderophores and requires PupB and two proteins homologous to  **FecI**  and  **FecR** .  Ref: 9148773 Arch Microbiol, 1997
- By analogy with the  **FecI**   **FecR**  FecA and PupI PupR PupB systems, our data suggest that bfrZ expression is inducible by binding of the cognate ligand to BfrZ and transduction of a signal through the envelope.  Ref: 11292812 J Bacteriol, 2001
- Transcription of the fec transport genes is positively regulated by  **FecI**  and  **FecR**  and negatively regulated by the Fe2 Fur repressor.  Ref: 7752886 Mol Microbiol, 1995
- Two regulatory genes,  **fecI**  and  **fecR** , located upstream of the fecABCDE transport genes, are required for induction.  Ref: 7752886 Mol Microbiol, 1995
- Five  **fecI**   **fecR**  iron receptor gene clusters from P. putida were also identified in the human pathogen Pseudomonas aeruginosa.  Ref: 12534467 Environ Microbiol, 2002
- In 11 cases, a  **fecR**  like gene was found adjacent to the  **fecI**  like gene and, in 10 cases, a gene encoding an iron receptor lies in the vicinity of the  **fecI**   **fecR**  cluster.  Ref: 12534467 Environ Microbiol, 2002
- Three specific proteins are involved FecA in the outer membrane,  **FecR**  in the cytoplasmic membrane, and  **FecI**  in the cytoplasm.  Ref: 12732308 Curr Opin Microbiol, 2003
